# ‘A lot of people think it’s just a Mickey Mouse role’: Role ambiguity among dementia support workers within secondary care and community hospital settings

**DOI:** 10.1177/14713012231220461

**Published:** 2023-12-15

**Authors:** Louise Margaret Prendergast, Ceryl Teleri Davies, Tracey Williamson

**Affiliations:** School of Medical and Health Sciences, 1506 Bangor University, Bangor, UK; 1507 Betsi Cadwaladr University Health Board, Bangor, UK

**Keywords:** dementia, secondary care, community hospitals, dementia support workers, activity coordinators, workforce, person-centred care, healthcare practitioners

## Abstract

**Purpose:** Dementia support workers (DSWs) are employed to improve the hospital care for patients living with dementia. An evaluation sought to understand the perspectives and experiences of DSWs and related healthcare practitioners within one health board, to identify any role ambiguity and inform future role development.

**Design/methodology/approach:** Framework analysis was used to synthesise data from semi-structured interviews and focus groups with dementia support workers, and a wider group of related healthcare practitioners.

**Findings:** Thirteen semi-structured interviews were conducted with DSWs. Two focus groups were held with DSWs (*n* = 2 and 4) and two with associated healthcare practitioners (*n* = 3 and 5). Participants described inconsistencies in the understanding and delivery of the DSW role. Role ambiguity was identified as a key theme.

**Originality/value:** This paper offers insight into challenges experienced by DSWs and addresses factors that could help improve and support the DSW role, and potentially the experience of other staff, and patients/people living with dementia. Overall, this evaluation highlights both the value of the DSW role in supporting the needs of patients/people living with dementia and the potential for person-centred activities to be used as therapeutic interventions.

## Introduction

There are more than 900, 000 people in the UK living with dementia, a figure expected to rise to 1.6 million by 2040 (Alzheimer’s Society, 2019). One in four hospital beds are occupied by someone with dementia (Lakey, 2009) and 42% of all acute hospital admissions for those over 70 are people living with dementia (Sampson et al., 2009). Within the NHS, dementia care is reported to be challenging due to workforce capacity issues and limited resources (Houghton et al., 2016; Turner et al., 2017). UK dementia services are described as fragmented (Martin et al., 2020) with a lack of specialist resources to provide proactive and preventative care within primary and community settings.

People living with dementia are more likely to be hospitalised due to their perceived needs and risks, primarily for co-morbid conditions (Alzheimer’s Disease International, 2016; Cunningham & Archibald, 2006). Many are admitted to hospitals without a formal diagnosis of dementia (Fogg et al., 2017; Timmons et al., 2016). Frequently admitted due to falls and dehydration (Timmons et al., 2016), patients with a cognitive impairment, including dementia, have a higher length of hospital stay than those who are not cognitively impaired (Fogg et al., 2017; 2018; King et al., 2006). This poses the risk of deconditioning and deterioration in cognitive function (Moyle et al., 2011) also referred to as ‘Pyjama paralysis’; the physiological change following inactivity during the hospital stay (Hoenig & Rubenstein, 1991). People living with dementia have a higher risk of experiencing poorer outcomes once in hospital: being more likely to experience episodes of delirium, and having higher rates of mortality (Fogg et al., 2017; Reynish et al., 2017). People living with dementia can experience fear and disorientation due to unfamiliar surroundings (Edvardsson & Nordvall, 2008), and may have difficulties in communicating their needs, with distress expressed through aggressive behaviours or refusing medication (Porock et al., 2015).

Improving dementia care in Wales and England have been key priorities. An objective for the UK National Health Service (NHS) is to improve the hospital experience for people living with dementia and their unpaid carers (NHS, 2019). The NHS Long Term plan (2019) aimed to reduce pressure on acute hospital resources, provide personalised care and give people more control over their own health to improve discharge processes. The Welsh Government Dementia Action Plan for Wales 2018–2022 aimed to embed a rights-based approach for the care of people living with dementia admitted to hospital. This included the implementation of John’s Campaign (Age UK, n.d) that recognised the importance of family carers being welcomed in hospital settings.

Evidence reflects that person-centred care (Kitwood, 1997) can benefit the overall experience and well-being outcomes for people living with dementia (Kim & Park, 2017; Tay et al., 2018). In a hospital setting, this is fostered by relationships with staff understanding and meeting the needs of people with cognitive impairment notably: attachment, comfort, identity, occupation, and inclusion (Clissett et al., 2014; Kitwood, 1997; Tay et al., 2018).

Nonetheless, providing person-centred care within acute or community hospital settings can be a challenge especially where co-morbidities, medical routines, and organisational efficiencies may be prioritised (Dewing & Dijk, 2016; Nilsson et al., 2013). Concerns have been raised around the quality-of-care people living with dementia receive in hospitals. Whilst the literature indicates that hospital staff recognise the importance of delivering person-centred care, within a busy ward environment they can feel restricted by the time allocated to support patients with dementia and limited in their understanding of the disease (Turner et al., 2017). Prioritising ward efficiency and reducing the perceived risks to patients impacts on provision of person-centred care (Featherstone et al., 2019).

There is a shortage of healthcare professionals (nurses and healthcare assistants) working with older people with dementia in hospital settings, due to factors including poor working environments leading to staff demotivation, affecting staff turnover and retention (Cheloni & Tinker, 2019). Whilst dementia care is valued as skilled work, there is variation in how hospital staff members conceptualise or value dementia care (Handley et al., 2019), and many staff lack the necessary skills and knowledge of dementia care (Schindel et al., 2016; Surr & Gates, 2017).

Recognising the challenging nature of the acute care environment for patients and staff, robust training, learning and development is needed (Surr and Gates, 2017), alongside person-centred care that involves the family/carers in decision making (Reilly & Houghton, 2019). Dementia support roles with this remit do exist for example, Dementia Champions and Dementia Specialist Nurses, found to contribute to improved outcomes for patients (Griffiths et al., 2015). Within Wales, Dementia Support Workers (DSW) are present within hospitals, to support people living with dementia within secondary care (Welsh Government, 2018).

At Betsi Cadwaladr University Health Board, Wales, a peer support network for DSWs was initiated in December 2021 and role ambiguity was quickly identified as a concern of the DSW community in the Health Board. Several titles were identified under the umbrella term ‘DSW’, including Activity Coordinators, Activity and Wellbeing Coordinators and Dementia Care Enablers. The core focus of DSWs is to promote meaningful activity and engagement with patients, to optimise person-centred care and to provide support for families including keeping socially connected to the patient. A lack of evidence relating to Health Care Assistant dementia support roles was identified in the literature searched, which this study sought to address. The aims of the evaluation were to identify and better understand the interpretation and delivery of the DSW role in community hospital and secondary care settings.

## Method

An initial scoping review of the literature and consultation with the newly appointed Consultant Nurse for Dementia at the Health Board guided this evaluation. Early engagement with DSWs about the proposed study design followed. A project steering group including the research team and Consultant Nurse, in consultation with DSWs facilitated discussion, problem solving and the co-production of the study throughout.

### Ethical considerations

Following correspondence with the UK Health Departments Research Ethics Service (NHS/HSC RECs) this study was considered a service evaluation and NHS ethics were not required. The study was conducted in line with Bangor University and other key ethics guidance (Social Research Association, 2021) with ethical approval gained from the Bangor University Healthcare Sciences (Post-reg) Ethical Review Panel (2022–17070). All prospective participants were provided with an information sheet by the Consultant Nurse which detailed the study and General Data Protection Regulation (GDPR) (2018) information. Participants were also provided with consent forms. Informed consent was provided verbally and recorded prior to the online interviews and focus groups. Upon consent, interviews and focus groups were recorded and transcribed verbatim by a GDPR compliant company. All identifiable data were removed from the data set and stored in separate password protected electronic files. Participants’ names and organisational details were replaced with codes in the transcripts.

Participants were given written and oral reassurances that there would be no repercussions or negative impacts on taking part in the study, and that confidentiality and anonymity would be maintained in the process and reporting of the study. All study information and data collection tools were bilingual (Welsh and English) and participants could partake in interviews and focus groups in Welsh, ensuring Welsh Language Standards Regulations were met throughout (Welsh Government, 2018). The consent process was active and revisited throughout each stage of the evaluation; participants were offered ‘free choice’ to participate without the fear of any perceived consequences if selecting not to participate. Participants’ identities remain unknown to the Consultant Nurse. The reporting of this study follows the Consolidated Criteria for Reporting Qualitative studies (COREQ) (Tong et al., 2007).

### Sampling and participants

The Consultant Nurse identified all potential study participants for the evaluation study, with purposive sampling then used by the research team to select participants from secondary and community hospital settings. The inclusion criteria were:

• DSWs from a broad range of locations across Betsi Cadwaladr University Health Board secondary and community care hospital settings.
• Other Betsi Cadwaladr University Health Board staff that work in secondary care or community hospitals for example Ward Manager, Matron, or other professionals in contact with DSWs undertaking their roles.

### Data collection

Data collection took place between March and August 2022. Qualitative methods were considered suitable for this evaluation, eliciting a comprehensive exploration of a topic under inquiry, from an in-depth perspective (Jamshed, 2014). Semi-structured interviews with DSWs were conducted to provide participants with the space and voice to share their perceptions and experiences of the DSW role (DeJonckheere & Vaughn, 2019; Wengraf, 2001). An interview schedule was developed from the literature and guided by the evaluation aims which included open ended questions relating to role, experience, ways forward and future development.

Focus groups, as group interviews (Kitzinger, 2005) were considered an appropriate method to stimulate group interaction and facilitate further discussion of experiences and views about the DSW role. Focus groups were held with DSWs, and wider healthcare staff that worked with DSWs. In a contained setting, it was anticipated that staff could feel empowered to deliver their views and experiences of working in shared roles. To attain focus and a safe space to encourage participation, focus groups were conducted separately with DSWs, and wider healthcare staff. Participants were asked to describe their own role, their experiences, and perspectives of the DSW role. Interviews and focus groups were conducted by the authors (Louise Prendergast and Ceryl Davies) who are experienced in interviewing. These were conducted online using Microsoft TEAMS, due to logistical ease, and where some Coronavirus restrictions remained within the hospital setting. Thirteen semi-structured interviews were conducted with DSWs, representing an 81% participation rate. Two focus groups were held with DSWs (*n* = 2 and 4) and two with associated healthcare practitioners (*n* = 3 and 5). Interviews and focus groups lasted for approximately 1 hour.

### Analysis

Data were analysed by the authors (Louise Prendergast and Ceryl Davies) using framework analysis, to enable comparison and contrasting data by themes, and across and within cases (Gale et al., 2013; Ritchie & Spencer, 1994). The following stages were followed:

• Data familiarisation: Transcripts from the interviews and focus groups were read and re-read .
• Developing a thematic framework: key themes from the interview and focus group schedules were used. Additionally inductive open coding of the transcripts ensured that any relevant codes were not missed.
• Indexing: relevant text was identified and marked with codes.
• Charting: indexed text was imported into Microsoft Word tables according to the themes.
• Mapping and interpretating the data: respondent checking was achieved through a knowledge exchange event to ensure details were accurate.

### Findings

This paper focuses on the following themes and subthemes derived from the analysis: Role clarity and ambiguity: ‘Lack of clear title and role definition’, ‘Role expectations’ including role boundaries, the perception of the role by others, and the need for training, and ‘Activities and resources’, including administration and documentation.

#### Role clarity and ambiguity

##### Lack of clear title and role definition

One of the key themes identified from the data was of inconsistencies in the title and role of the DSW, and associated outcomes. DSWs reported that several titles were used to describe their role; within job descriptions and used by other staff. These included ‘Dementia Care Enabler’, ‘Dementia Support Worker’, ‘Activity Coordinator’, ‘Dementia Activity Worker’, and ‘Senior Healthcare Worker’. The title ‘Dementia Nurse’ was referred to, however, it was noted by two participants (one healthcare practitioner and one DSW) that the role was not attached to any nursing qualifications. ‘Dementia Champions’ was also mentioned, although as one DSW stated, this did not reflect the role recognised by the Alzheimer’s Society. Different titles were said to reflect the different role expectations between hospital settings:

There are so many differences between dementia support where there’s different names to start off with. So, I’m a Dementia Support Worker, but in other places it’s an Activity Coordinator. There’s something else somewhere else as well. So, you haven’t got a consistency with what the name of the role is. (DSW-2, interview)

A small number of DSWs expressed that having the term ‘dementia’ in the title was problematic, regarded as potentially causing alarm to a patient or their family/informal carer. DSWs reported that many patients did not have a formal dementia diagnosis or did not wish to be associated with the term ‘dementia’:

I always say I’m the activity lady because I have the people that are poorly saying, “Oh no, love. I’ve got no dementia. I don’t need you.” That’s not what I’m there for. I’m there for everybody. So, I just say, “I’m the activity lady. If you need me, I’m here.” (DSW -8, interview)

A similar sentiment was reflected by some healthcare practitioners who suggested that the wider benefits of the role could be reflected in the title:

We put all our jobs out as activity/dementia support workers so that they’re not specifically earmarked to work with just dementia patients. It’s all the patients on the ward. (Healthcare practitioner, focus group 3)

##### Role expectations

All participants understood that the role of the DSW was to support patients ensuring their hospital stay was as comfortable as possible; to prevent boredom and distress and pass on relevant information to other staff. Other healthcare practitioners recognised the importance of delivering person-centred care in an otherwise medicalised setting:

I think they’re really, really important roles ‘cause being able to give some tailored, focused attention, to be alongside the person with dementia, to help distract, to meet their needs and to tailor an approach, rather than having the focus on care that’s being provided. (Healthcare practitioner, focus group 2)

Nonetheless, DSWs and other healthcare practitioners expressed that they had no clear understanding or expectations of the role. Some DSWs reported that they were not aware of the implications of the role prior to starting the job, and were expecting to adhere to pre-planned activities:

My perception of how that was going to work before I started the role is different to what the reality of it is. So, I thought I’d be doing, you know, 2 o’clock on a Monday we’d be doing crafts, and then 2 o’clock on a Tuesday we’d have coffee and a film, and that’s just not the reality. It’s not a possible thing. (DSW, focus group 1)

Healthcare practitioners recognised the DSW role had no clear definition and evolved over time, adapting according to patient needs and the hospital context:

There’s been no real clear definition or direction about what the role is. And I think as the role was introduced, different areas, different hospitals have had to sort of work that person to adapt that role, innovate that role to different situations. (Healthcare practitioner, focus group 3).

A lack of clear expectations of the role led to some DSWs admitting that it was some time before they were aware of the impact of their work, and whether they were doing the job correctly:

It took me a good few six months to realise that, am I really doing any good? Is this what they’re expecting of me? The expectation, I think, is not there. (DSW-8, interview)

With DSWs highlighting that initially, they were unsure what exactly was expected of them. they referred to ‘working it out themselves’ and ‘being in at the deep end’. A lack of initial support was reported by one DSW as a probable explanation for some DSW leaving the role soon after starting:

More support right at the beginning of the role because I think that’s one of the major issues why so many people leave the role early on. (DSW-2, interview)

##### Role boundaries

Participants reported that DSWs were frequently expected by staff to deliver personal care to patients on the wards. Tasks included escorting patients to the toilet, washing them, and assisting with meals:

I know some of them do do personal care, but basically on a ward like this, all you’d do all day would be taking people to the toilet all day. (DSW-5, interview)

Providing personal care was reported to impact on delivering person-centred care and getting to know the patient:

If you do personal care, you can get dragged in to doing that all day, every day, and therefore that doesn’t release the time to do what the role entails. (DSW, focus group 1)

This expectation was seen as due to a lack of clear role definition, and related role boundaries. Both DSWs and healthcare practitioners acknowledged a need to address role expectations and role boundaries, to all staff including DSWs:

So, there’s something that’s got to be where they turn around and say, “Right, okay. This is the job.” It’s got to be explained to everybody. (DSW-8, interview)

Nonetheless, there was a consensus among DSWs and health practitioners that providing additional support on the ward, including personal care was justifiable where there were issues of staff shortages, as patient safety was paramount:

When you’ve got staffing shortages on wards and you’re pulling one staff from one area to another to mitigate the risk, you look at everybody that’s on that ward. You’re looking at student nurses. You’re looking at health teams. You’re looking at extra support workers. It’s all there on safe care. (Health practitioner, focus group 3).

##### Perception of the role by others: Not feeling valued – a ‘Mickey Mouse’ role

Whilst no Healthcare practitioners we spoke to referred to the DSW role as easy and/or something that anyone could do, many participants reported that they believed others held the view that the DSW was a ‘Mickey Mouse’ role (defined as: ‘silly, childish, easy or worthless’: (Collins English Dictionary, no date)

A lot of them think it is a Mickey Mouse role and you get it really easy, especially ‘cause I’m left to my own devices. But other times, it’s quite frustrating because you’re asked to be doing stuff when you’re already doing stuff. (DSW-7, interview)

This perception was linked to some staff not only misunderstanding the role of the DSW, but not understanding dementia, and the purpose and effectiveness of person-centred care:

It’s perceived as an easy sort of futile job role, but the staff don’t get it. I don’t think they understand the role. (DSW-1, interview)

Quite a lot of people, staff I mean as well, they’re just like, “Oh, she does activities.” Well, I’m a bit more than that, personally. (DSW-12, interview).

Subsequently, several DSWs reported feeling demotivated, and/or feeling guilty when engaging with patients and attending to their individual needs which was interpreted as ‘frivolous’ and not necessary:

I think a lot of people see it as something surplus, as something extra, as something that is frivolous. And I believe that it is particularly difficult to come to people who are on a lower paygrade. To be fair to them, what they see is a person coming in who just does these extra things when they do the work of making sure they are clean and have food and everything. And I’ve had people say things like that to me too. One person came to me the other day, “Oh, I’m dying to come in and do everyone’s nails.” At the time I was just gob smacked. (DSW-11, interview)

Educating other staff on the purpose of the DSW role could aid their understanding, and it was proposed that this could prevent the ramifications of these perceptions often keenly felt by the DSWs:

Other staff just thinking that you’re basically coming and just sitting with patients all day and not actually doing any work, which puts a negative connection between you and other staff really. So maybe it would be beneficial for other staff to understand what the actual role is and know what we’re there for really (DSW-2, interview)

##### Need for training

DSWs reported that they came to the role with minimal formal training. All reported undertaking mandatory online training. However, many referred to their previous personal and/or professional knowledge of dementia to inform their practice:

It was literally just like, “There you go.” And I didn’t get any training at the beginning either. (DSW-1, interview)

No training. I had a friend that had dementia, so I kind of worked with the activities. That was no problem. The only elderly I’ve had is my activity coordinator I did in a nursing home. (DSW-8, interview)

Motivated to provide a better experience for patients and to underpin their confidence in the delivery of support, many DSWs highlighted their wish for additional training:

I would be more confident, maybe having done manual handling earlier in the training, maybe I would be more confident. (DSW-11, interview)

However, one DSW stated that they felt supported by their manager in areas of self-development:

I was given the time off by my manager. I was obviously paid for being there. I was given the train fare back to get there. So, yeah, I’m wholly supported in self-development. (DSW-7, interview)

According to the DSWs, support had improved with the introduction of monthly meetings facilitated by the recently instated Consultant Nurse for dementia. In providing a space for shared practice, the meetings enabled DSWs to discuss their concerns or requirements:

I think that’s going to be a monthly thing now with other ones, and I found that really helpful. And I think we were all sort of requesting some sort of formal training. (DSW-4, interview)

#### Activities and resources

All participants acknowledged that the DSW role required knowledge of dementia, skills and time to attend to and deliver activities that distracted, calmed, and soothed the patient. In delivering person-centred support and as a therapeutic intervention, this could reduce the need for medication not scheduled and on request (PRN):

You’ve reduced distressed behaviour; you’ve reduced the need for PRN medication. And it’s just a different way of approaching the task, which is why I think that it’s therapeutic rather than caregiving (Health practitioner, focus group 4)

Healthcare practitioners considered DSWs as instrumental in supporting patients, initiating, and maintaining exercises as directed by their Occupational or Physio Therapists. DSWs spoke of being well placed to integrate exercises with activities and shifting the perception of exercises from being medicalised to being fun:

The physios watched because they say when I do a fun thing, a game, they (the patients) want to compete, like the Olympics, whereas when they (the Occupational or Physio Therapists) ask them to raise your hand 10 times or whatever, you’ve got no chance - cos it’s not good, not fun. (DSW-8, interview)

Healthcare practitioners referred to opportunities for learning from DSWs, and many shared the view that the DSW role could be situated with line management/supervision from Occupational Therapy. This was perceived to bring longer term benefits in reducing acuities:

It’s therapeutic, and, you know, home in on that. And then that will reduce acuity in time and so forth. And it’s all part of that multidisciplinary approach. (Healthcare practitioner, focus group 3)

To facilitate person-centred care tailored to an individual’s preferences, DSWs acknowledged the need for resources, for example, puzzles and craft materials. The majority stated that they did have access to a range of equipment and activities:

And I’ve got games. I’ve got books. I’ve got things like Connect 4. We’ve got Snakes and Ladders, games like that, which is really nice. I’ve got books with pictures in as well, big pictures, so they can look at them. We’ve got loads of things. (DSW-3, interview)

However, budgetary constraints and limited access to funding were recognised by some participants as impacting on their resource availability, and the subsequent the delivery of activities:

I can’t do my role of doing the activities without having the stuff there to do it really. (DSW-2, interview)

A day room was believed to be an essential resource. This space conferred many benefits; from holding a shared activity for patients, to providing a quiet space to reduce anxiety levels that could be worsened by the busy ward environment:

Her anxiety levels were all over the shop. So, the fact that I can go down to the day room and close the room means I can take those things away. (DSW, focus group 1)

In many cases, there was no day room where patients could be taken away from ward environment, and as such, a separate space was one of the key recommendations made:

The ward is really busy and chaotic […] quiet time with the Dementia Support Worker or our own Support Workers; there just isn’t anywhere at all. We don’t have anything. When we’ve had games and things like that in the past, it’s been things the Dementia Support Workers have brought with them. So, we don’t have any resources here at all really, which is a real shame. (Healthcare practitioner, focus group 4)

#### Administration and documentation

To achieve a better understanding of the patient, their personal preferences, likes and dislikes, DSWs referred to administering documentation they commonly used. The ‘This is me’ tool, developed by Alzheimer’s society (Alzheimers.org.uk, n.d) was referred to by all. Not only did this enable the DSW and additional staff to have a greater insight into the patient, the task of filling in the documentation could help the DSW foster a good rapport with the patient and/or their family:

[This is me] it’s really helpful because I think it’s important to find out what the person likes, what they enjoy, makes them happy. You know, if they’re upset, what can I do, what can staff do to help them. It’s just a massive help. You get to know them a lot better as well. (DSW-3, interview)

‘This is me’- especially patients with advanced dementia, you make a really good rapport with the relatives, which I think is important (DSW-1, interview)

However, as not all patients had a formal diagnosis of dementia, this tool was not always perceived to be appropriate:

It’s not often that I’ll actually approach a person with a diagnosis of dementia and say, “Oh, do tell me about your dementia,” because you wouldn’t do it with any other diagnosis. It’s the elephant in the room, but at the same time, it’s not that big a deal that we have to just emphasise it. So I’ve never done a ‘This is me’ with a patient. (DSW-7, interview)

More suitable for a wider range of patients, a document known as ‘About me’ was referred to, contained questions less specific to dementia. Although often used by those who knew about it, there was limited awareness of this document, and those that were aware of it had found it serendipitously, for example, through an online group meeting:

I think that it’s a really handy tool to have even if there’s no official diagnosis, even if it’s just, you know, people who are a bit confused or have got a UTI […] I didn’t know about it (‘About me’) until I had the DSW group TEAMS thing the other week. (DSW-4, interview)

To complete administrative tasks and paperwork, DSWs noted that they required access to a computer or laptop. Reflecting the limited resources available to them, and the need for them to work flexibly, many remarked that they gave other staff priory over the use of computers:

I haven’t got my own laptop or my own computer. So, like I say, I try and get my work done in the morning so they can use it in the afternoon […] I have to use ones in the bay. But as I say, if they want to come and use the computer, I’ll come off it. (DSW-9, interview)

## Discussion

It is widely reported that the delivery of person-centred care can confer benefits to people living with dementia generally (Manthorpe & Samsi, 2016) and in hospital settings (Tay et al., 2018). Less is known about those who work in roles specifically designed to deliver person-centred care within acute and community hospital settings, where time pressures and clinical needs can result in task orientated care being prioritised over individualised care (Cowdell, 2010). This evaluation sought to explore the perspectives and experiences of DSWs, within acute and community hospital settings, and related healthcare practitioners. A number of interrelated themes were evident from this evaluation. A key theme identified was a lack of role clarity, pertaining to having clear information about role expectations (Mayrhofer et al., 2016), with implications on the wider expectations and perceptions of the role by DSW and other ward staff. Role ambiguity was additionally identified; characterised by unclear expectations of the job and a lack of information needed to undertake it (Dailey, 1990). Cengiz et al. (2021) report commonly defining attributes of role ambiguity in their (concept analysis) literature review on hospital nursing staff. The authors identify a lack of information and/or clarity of the goals, objectives and responsibilities of the role, and unpredictability of the consequences. The antecedents of role ambiguity are identified as a lack of clear role definition, lack of education and training, challenges in communication, supervisory support, and behaviours. Similarly, impacts of role ambiguity include job dissatisfaction, burnout, and intent to leave. The themes derived from this evaluation and described in this paper can be clearly mapped onto these antecedents (see Table 1).

**Table 1.** Theme map.

Antecedents of role ambiguity (Cengiz, 2021)	Evidenced in evaluation study	Implications	Recommendations
**Lack of clear role definitions:** job descriptions, objectives responsibilities and expectations	Variations in role to the agreed responsibilities outlined in the job description	Misrepresentation and misunderstanding of job role and responsibilities.	Standardised role description that encompasses role differences e.g., location and flexibility to deliver person-centred care
	Differences in title	DSW are pulled away from core activities to provide wider support	Avoid staff shortages to entail role delivery not distracting from patient
	Title does not reflect activities undertaken with all patients	Not all patients have a dementia diagnosis	Clearer role definitions supported by senior figures
			Title and role to reflect activities not (just) dementia diagnosis
**Lack of education/training: **prior to starting the role	No consistency of training, baselines, tools, or standards reported	DWS feel ‘thrown in’ and lack confidence in some areas	Induction sessions at start of job
	Learning on the job or from previous personal/professional experience	Some DSWs leave role early on	Shadowing and mentoring
			Opportunities for additional dementia training
**Communication problems:** not all tasks are explained or responsibilities communicated	Not all staff understand the therapeutic benefits to patients following DSW intervention	DSWs feel undervalued	Continue monthly meetings with consultant nurse to share concerns
	Some ward staff are unaware of the tasks and benefits related to the DSW role	DSWs pulled into additional tasks	Communicate the role to others including importance of person-centred care
**Supervisory behaviours and support:** lack of leadership and continuous support from line manager and co-workers	Support from co-workers on ward lacking where role not understood and negative perceptions received	DSWs feel guilty or demotivated	Continue monthly meetings with consultant nurse to share concerns
			Ensure the role is widely understood by all, and outcomes from delivering person-centre care
**Lack of information (information deficiency**) Not knowing what is expected from the role	Limited standardisation of documentation	DSWs adopt flexible and unpredictable working based on available resources	Regular feedback on performance to DSWs
	Limited access to computer for administrative tasks	Resource constraints (day room and activities) potentially impact on patient’s experience and wellbeing	Improve access to technology for administrative tasks
	Lack of budget/separate room access		Review access to equipment budgets
**Unpredictability** lack of feedback	Role is unpredictable (expectations vs. reality)	Flexibility centred on patient with supported autonomy within role	Regular feedback on performance for DSWs
	DSW question if they are doing well		More peer-to-peer networking to identify and exchange good practices

Role ambiguity underpinned by staff misunderstanding and miseducation, a lack of training opportunities, poor communication and support can impact on role delivery including lower motivation and reduced job satisfaction. Similarly, Handley et al. (2019) found that dementia care was valued differently by hospital staff– with some ward staff not recognising it as skilled work: “I don’t think it’s a skill, you just ensure that you don’t let them fall, that’s it.” (Healthcare Assistant in Handley et al., 2019, p. 68). Kahn et al. (1964) noted that role ambiguity results in ‘tension, job dissatisfaction, a sense of futility, and lower self-confidence’. Indeed, attrition and staff turnover are high in the dementia care workforce with a lack of role recognition and feeling valued being indicated as contributory factors (Hale et al., 2021). With a growth in people living with dementia, the recruitment and retention of Dementia Support Workers is crucial. Where dementia care within the hospital is valued as skilled work by the wider healthcare staff, this could contribute to greater job satisfaction to the dementia care workforce (Handley et al., 2019).

Biopsychosocial approaches to dementia care reportedly deliver positive experiences and outcomes for patients with dementia, their unpaid carers and the wider healthcare staff who may feel more competent and confident in their role (Aldridge et al., 2020). Where patients with dementia may resist medications and food and drink, their health can deteriorate leading to reduced mobility, increased dependence and poorer outcomes and experiences (Featherstone et al., 2019). The role of the DSW is to deliver person-centred care; attend to patient’s psychological needs and potentially improve their hospital experience, receptiveness to medical and/or therapeutic interventions and improve their overall wellbeing outcomes.

Although more time intensive, where person-centred care is delivered, patients may be less likely to display responsive behaviours (Gwernan-Jones et al., 2020), and medication potentially reduced (Banks et al., 2014). The term ‘threshold concept’ (Meyer & Land, 2003) is used by Wilkinson et al. (2016) to describe how person-centred care, when delivered in acute settings, can lead to time saved in the long run.

Globally, the impact of dementia brings significant social and economic costs (Alzheimer’s Disease International, 2016; WHO, 2021), indicating the importance of addressing the growing need and impact of dementia. International evidence highlights the association between positive staff attitudes and better quality of care for PLWD (for example, Schneider et al., 2021; Travers et al, 2013). Improving access to training, staff support and mentoring for all health and social care staff supporting PLWD will assist in supporting the development of delivering person-centred care, which is crucial in elevating the impact of dementia (Mustafa, et al., 2013).

## Recommendations

Role recognition can be supported by endorsement from management and senior clinical leaders (Handley et al., 2019). This can also contribute to the confidence of DSWs to have the authority to support patients’ personalised needs (Abbott et al., 2021). Hospital staff can learn from the specialised skills and knowledge of health carers working with people living with dementia, to help them to develop their own practice and support patients who are distressed (Cheloni & Tinker, 2019). Furthermore, accreditation training schemes can contribute to career progression of the dementia care workforce, and reinforce the understanding of the value and skills of dementia care (Handley et al., 2019).

Good relationships between the nurse and manager are reported to reduce role ambiguity (Cengiz et al., 2021). Our findings suggest some role ambiguity was mitigated by the provision of a Consultant Nurse in improving communication, highlighting the role of the DSW to, and facilitating improved relationships with other staff. Nonetheless, the Consultant Nurse as a ‘change agent’, needs to be accompanied by awareness of contextual factors: organisational endorsement of dementia care in general and understanding of what constitutes good person-centred care. Whilst a recommendation could be to standardise some aspects of the DSW title and role as a hospital wide approach, the flexible and creative element of the role must be retained to facilitate person-centred care where there is no one size fits all. An understanding of the contextual factors specific to each hospital setting will impact on how they accommodate and embed any new practices (Godfrey et al., 2018). Furthermore, managing risk within a hospital setting is a major factor that impacts on the time and resources available to staff, therefore ideally, roles and role clarification need to be addressed before staffing resources are implemented (Handley et al., 2017).

## Study limitations

This evaluation provided a unique opportunity for the voices of Dementia Support Workers to be heard. However, this was limited to eligible participants within a single Health Board. We also acknowledge the limits to the broader applicability of the findings from related healthcare practitioners, as staff within other areas may have different perspectives and experiences.

Potential participants were identified by the Consultant Nurse for dementia (Tracey Williamson); hence we recognise that participants may have potentially been reluctant to express negative feelings. However, participants were given reassurances that there would be no negative impacts on them by taking part in the evaluation. This evaluation was small-scale in nature and based in one Health Board and is not intended to be representative of the UK or indeed Wales as a whole. Despite purposively selecting a sample to include a diverse range of participants, the total sample represented 81% of participants invited to participate. As illustrated, strong patterns emerged in the views of the DSW. Whilst the sample size was small, no new themes were evident in the final few interviews, and we were satisfied that data saturation had been met.

## Conclusion

With a changing demographic reflecting increases in population ageing, more people will present to acute care settings with co-morbidities, including dementia and cognitive impairment. Further research is required to revisit the same site to establish the longer-term evolution of the DSW role and to revisit the themes related to role ambiguity. Furthermore, the continuity of care following hospital discharge to the community DSW could be investigated. The wider impact and outcomes of the DSW role for related stakeholders including healthcare practitioners, patients and their informal carers/family are unknown, therefore further exploration, is warrented to capture the wider social and economic value of the role.
